# Quantifying cilia beat frequency using high‐speed video microscopy: Assessing frame rate requirements when imaging different ciliated tissues

**DOI:** 10.14814/phy2.15349

**Published:** 2022-06-08

**Authors:** Luke Scopulovic, Deanne Francis, Elvis Pandzic, Richard Francis

**Affiliations:** ^1^ Cilia Research Laboratory, College of Public Health Medical and Veterinary Sciences James Cook University Townsville Queensland Australia; ^2^ Biomedical Imaging Facility University of New South Wales Sydney New South Wales Australia

**Keywords:** cilia, cilia beat frequency, ependyma, respiratory epithelium

## Abstract

Motile cilia are found in numerous locations throughout our body and play a critical role in various physiological processes. The most commonly used method to assess cilia motility is to quantify cilia beat frequency (CBF) via video microscopy. However, a large heterogeneity exists within published literature regarding the framerate used to image cilia motility for calculating CBF. The aim of this study was to determine the optimal frame rate required to image cilia motility for CBF assessment, and if the Nyquist theorem may be used to set this rate. One‐second movies of cilia were collected at >600 fps from mouse airways and ependyma at room‐temperature or 37°C. Movies were then down‐sampled to 30–300 fps. CBF was quantified for identical cilia at different framerates by either manual counting or automated MATLAB script. Airway CBF was significantly impaired in 30 fps movies, while ependymal CBF was significantly impaired in both 60 and 30 fps movies. Pairwise comparison showed that video framerate should be at least 150 fps to accurately measure CBF, with minimal improvement in CBF accuracy in movies >150 fps. The automated script was also found to be less accurate for measuring CBF in lower fps movies than manual counting, however, this difference disappeared in higher framerate movies (>150 fps). In conclusion, our data suggest the Nyquist theorem is unreliable for setting sampling rate for CBF measurement. Instead, sampling rate should be 3–4 times faster than CBF for accurate CBF assessment. Especially if CBF calculation is to be automated.

## Graphical abstract

The aim of this study was to determine the optimal frame rate required to image cilia motility for cilia beat frequency assessment, and if the Nyquist theorem may be used to set this rate. our data suggests the Nyquist theorem is unreliable for setting imaging sampling rate for cilia beat frequency measurement. Instead, sampling rate should be 3–4 times faster than cilia beat frequency for accurate CBF assessment.

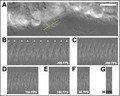

## 1 INTRODUCTION

Cilia are hair‐like projections arising from the cell membrane that extend into the extracellular environment (Satir & Christensen, 2007). Cilia appeared at the beginning of eukaryotic cell evolution ~1 billion years ago (Satir et al., 2008), and have since evolved to perform a wide range of physiological functions (Anvarian et al., 2019; Wallmeier et al., 2020). Motile cilia that beat in a coordinated manner are found in numerous locations throughout vertebrate bodies and play critical roles in many physiological processes including the propulsion of mucus across airway epithelia (Legendre et al., 2021) and the propulsion of cerebral spinal fluid within the brain ventricles (Kumar et al., 2021).

The most used method to assess motile cilia function is to quantify cilia beat frequency (CBF) via high‐speed video microscopy (Reula et al., 2021; Rubbo et al., 2019; Shapiro et al., 2018). However, a large heterogeneity exists within the published literature as to what frame rate per second (fps) to use when imaging cilia motility for subsequent CBF calculation; with published frame rates varying between 28–500 fps (Abdelhamed et al., 2018; Abdelhamed et al., 2020; Bustamante‐Marin et al., 2019; Chen et al., 2016; Hagiwara et al., 2020; Liu et al., 2021; Mateos‐Quiros et al., 2021; Mikhailik et al., 2021; Reula et al., 2021; Smith et al., 2012; Zahid et al., 2020). The large variation in sampling rates suggests some studies may be under‐sampling or oversampling cilia motility, which could result in inaccurate quantification of CBF or generation of large volumes of oversampled data requiring analysis and storage. The lack of a standardized motile cilia imaging protocol is problematic because it hinders high‐speed video microscopy from being clinically adapted as a diagnostic tool to help detect motile cilia diseases, such as primary ciliary dyskinesia (Bricmont et al., 2021; Shapiro et al., 2018).

Determination of the optimum sampling rates for cilia video microscopy is also becoming increasingly important with the rapid advancements in digital camera technology (Liang & Wang, 2021; Pauls & Mukasyan, 2017). This has put high‐speed digital imaging within the budget of even the most modest of laboratories, with even consumer‐grade devices (e.g., iPhone) now having the possible frame rates required for cilia imaging (Chen et al., 2016; Mateos‐Quiros et al., 2021). Determining the optimal sampling rate for cilia video microscopy is needed due to the dramatic increase in imaging rates seen in the latest generation of scientific high‐speed video cameras (Etoh et al., 2013), where fps oversampling can greatly increase file sizes needing to be processed and archived (Aiello et al., 2019; Yaffe, 2019).

Thus, the aim of this study was to determine optimum video framerates when imaging different ciliated tissues beating at different rates for subsequent CBF calculation. To achieve this, different motile cilia populations were imaged at >600 fps to generate movies of cilia displaying a wide variety of innate CBFs; these movies were then down‐sampled to generate new movies of varying framerates (30–300 fps) to allow CBF to be quantified for identical cilia but at different framerates. The effect of movie fps on CBF calculation was first assessed by comparing CBF means between different ciliated tissues incubated at different temperatures to determine optimal movie fps for each ciliated population. CBF data from all tissues were subsequently combined into a single dataset, and pairwise comparisons were made between reference CBFs (CBFs calculated at 300 fps) and calculated CBFs for the same cilia but at different sampling rates allowing for a more direct delineation of the relationship between movie fps and CBF measurement accuracy. Finally, all CBF data was analyzed using two different CBF quantification methods (manual counting, or MATLAB automation) to determine if quantification method influenced the observed fps‐CBF relationship.

## 2 METHODS

### 2.1 Animals

All animal procedures were conducted in accordance with the James Cook University Animal Ethics Committee (Ethics# A2783). C57/BL6 mice of mixed sex and age destined for euthanasia during routine colony maintenance were donated to this study by the Australian Institute of Tropical Health & Medicine small animal colony.

### 2.2 Ciliated epithelia isolation and preparation

Ciliated epithelia samples were stored and imaged in L‐15 media lacking phenol red (ThermoFisher Scientific; 21,083,027) and supplemented with 10% FBS (ThermoFisher Scientific; 16,000,036), 100 units/ml of penicillin G sodium, and 100 μg/ml of streptomycin sulfate (ThermoFisher Scientific; 15,140,122).

To image respiratory cilia, mice were euthanized via CO_2_ asphyxiation and trachea were immediately isolated and placed into L‐15 media on ice before being prepared for video microscopy as previously described (Francis & Lo, 2013). In brief, trachea segments 3–4 tracheal rings in length were cut longitudinally through the segment's anterior aspect, then longitudinally through the middle of the trachealis muscle using micro dissection scissors, leaving two sections of equal‐sized trachea tissue suitable for imaging (Francis & Lo, 2013). The trachealis muscle when cut longitudinally displays a propensity to curl upon itself presenting an unimpeded sagittal view of the respiratory epithelia, allo wing clear visualization of ciliary motion along the entire length of each tracheal segment (Movie S1A,B). Each trachea section was mounted with a few drops of L‐15 media in an imaging chamber constructed using two 24 × 50 mm #1 coverslips (Bio‐Strategy; EPBRCS24501GP) sandwiching a 0.254 mm thick layer of silicone sheet (AAA Acme Rubber Co; CASS‐.010X36‐64,909) from which a central square had been cut to form a shallow walled imaging chamber.

To image ependymal cilia, the head of each mouse was placed into a 60 mm culture dish on ice with L‐15 media; the calvaria was removed using sharp dissecting scissors and the intact brain was gently lifted out of the cranial cavity using blunt forceps. Brains were sectioned fresh in a sagittal mouse brain matrice (Electron Microscopy Sciences; 69,090‐S) filled with 4°C L‐15 media. Brain sections (0.5 mm thick) were then placed into 35 mm culture dishes filled with 4°C L‐15 media and the brainstem and cerebellum were removed using blunt dissection. Ependymal cilia within the third ventricle (Figure S2) were imaged in a similar manner as the airway cilia, by mounting brain sections with a few drops of L‐15 media into imaging chambers constructed using two 24 × 50 mm #1 coverslips (Bio‐Strategy; EPBRCS24501GP) sandwiching a 0.51 mm thick layer of silicone sheet (AAA Acme Rubber Co; CASS‐.020X36‐64,909) from which a central square had been cut (Francis & Lo, 2013) (Movie S1C,D).

### 2.3 Imaging cilia motility

Cilia were imaged using a Zeiss Axiovert 200 microscope with a 63×/1.4 Oil objective (Zeiss; 420,782–9900), Immersol 518F Immersion Oil (Zeiss; 444,960–0000), and DIC microscopy. To maintain samples at 37°C the microscope was fitted with a custom polycarbonate incubation chamber that enclosed the microscope stage, objectives, and condensers; the incubation chamber was fitted with an IncuStat Advanced incubator control module (Incubator Warehouse; PTST) and two IncuStat heaters placed either side of the stage. Chamber temperature was monitored using digital thermometers placed above and below the microscope stage.

Recordings of cilia motility were collected using a high‐speed USB3.0 digital camera (ProSciTech; EM101500A), with a 1.5MP 1/2.9" Sony Exmore CMOS sensor, connected to a PC running Windows 10 and ImageView software (ImageView, version ×64 4.7). Lengths of ciliated epithelia were orientated horizontally within the cameras field of view by rotating the camera on its C‐mount, this allowed 1 second movies to be collected at >600 fps with a 1440 × 240 pixel image size corresponding to a 150 × 25 μm field of view (0.104 μm/pixel).

To obtain a wide spread of possible CBFs, samples of airway and ependymal cilia were imaged at either room temperature or 37°C. One‐second movies of cilia motion were collected at >600 fps and saved as AVI movies. FIJI (ImageJ v2.1.0/1.53c) (Schindelin et al., 2012) was then used to convert initial recordings into a set of movies of the same field of view with varying framerates (300, 200, 150, 100, 60, and 30 fps). Kymographs of cilia motility were generated from each movie in ImageJ by using the line tool to draw a line through the cilia of a single ciliated cell (Figure S1A). This line was then ‘Resliced’ in ImageJ to generate a kymograph image (Figure S1B). Identical lines were applied to each movie with the same field of view but different framerate using the ImageJ command ‘Restore Selection’, this generated kymographs for the same cilia at differing framerates (Figure S1B–G). An average of three cilia kymographs, representing the ciliary motion of three different ciliated cells, were collected randomly per set of movies. CBF was subsequently quantified from the kymographs using two different techniques. Firstly, CBF was manually calculated by opening kymograph files in image processing software (Photoshop CS6 or GIMP v2.10), and averaging the number of pixels between each kymograph wave peak, from which CBF was determined using the following equation:

$$\mathrm{CBF}\;\left(\mathrm{Hz}\right)=\frac{\text{Original movie framerate}\;\left(\mathrm{fps}\right)}{\text{Average number of pixels between wave peaks}}$$

Secondly, CBF was calculated from the same kymograph images using a custom MATLAB script (MathWorks Inc, version 9.9.0, R2020b), a link to the script used is available online (https://github.com/ElvPan/CBF‐analysis). In brief, a temporal spectrum for the pixels in each kymograph was computed using the fast Fourier transform (FFT) algorithm. The dominant frequency (frequency with the highest peak) was then identified using the MATLAB function ‘findpeaks’ which corresponded to CBF for that sample (Awatade et al., 2021). A user‐friendly GUI and complete guide for running this automated CBF analysis is also available online (https://github.com/ElvPan/CBF‐calculator).

### 2.4 Data analysis and statistics

CBF data was analyzed in two different ways. Firstly, the CBF was averaged for each tissue, temperature, and quantification method; resultant means were then compared using two‐way ANOVA and post‐hoc Šídák's multiple comparisons test (Prism 9, GraphPad Software). Secondly, the CBF data from all tissues and temperatures at 300 fps was combined into a single CBF dataset. CBFs calculated from the 300 fps movies was then defined as the reference CBF (assumption being that CBF calculated from the highest sampled movies being the most accurate). The reference CBF value for each cilium was then compared in a pairwise fashion with its CBF calculated from the movies with lower framerates. This pairwise comparison was conducted on reference CBFs beating between 0 and 15 Hz, reference CBFs beating between 0 and 30 Hz, and reference CBFs beating between 0 and 50 Hz. These three groups of beat frequencies were chosen to aid in delineating the relationship between movie fps and CBF measurement accuracy. Linear regression analysis was used to calculate best‐fit lines and R^2^ values for each pairwise comparison dataset (Prism 9, GraphPad Software) then ANCOVA was used to compare best fit lines with an idealized dataset where calculated CBF perfectly matched reference CBF for each cilium (i.e., slope = 1). Significant divergence of best fit lines from this idealized dataset suggesting significant impairment in the accuracy of measuring CBF at that framerate.

## 3 RESULTS

### 3.1 Frequency distribution and mean CBF values of sample data

Tissue from a total of 23 mice were imaged. This included tissue from 13 airways and 8 brains imaged at room temperature (~24°C), and tissue from 10 airways and 9 brains imaged at 37°C. The tissues and temperatures used in this study were selected to provide a dataset with as much variation in CBF as possible to better assess the relationship between imaged movie fps and calculated CBFs.

**FIGURE 1:**
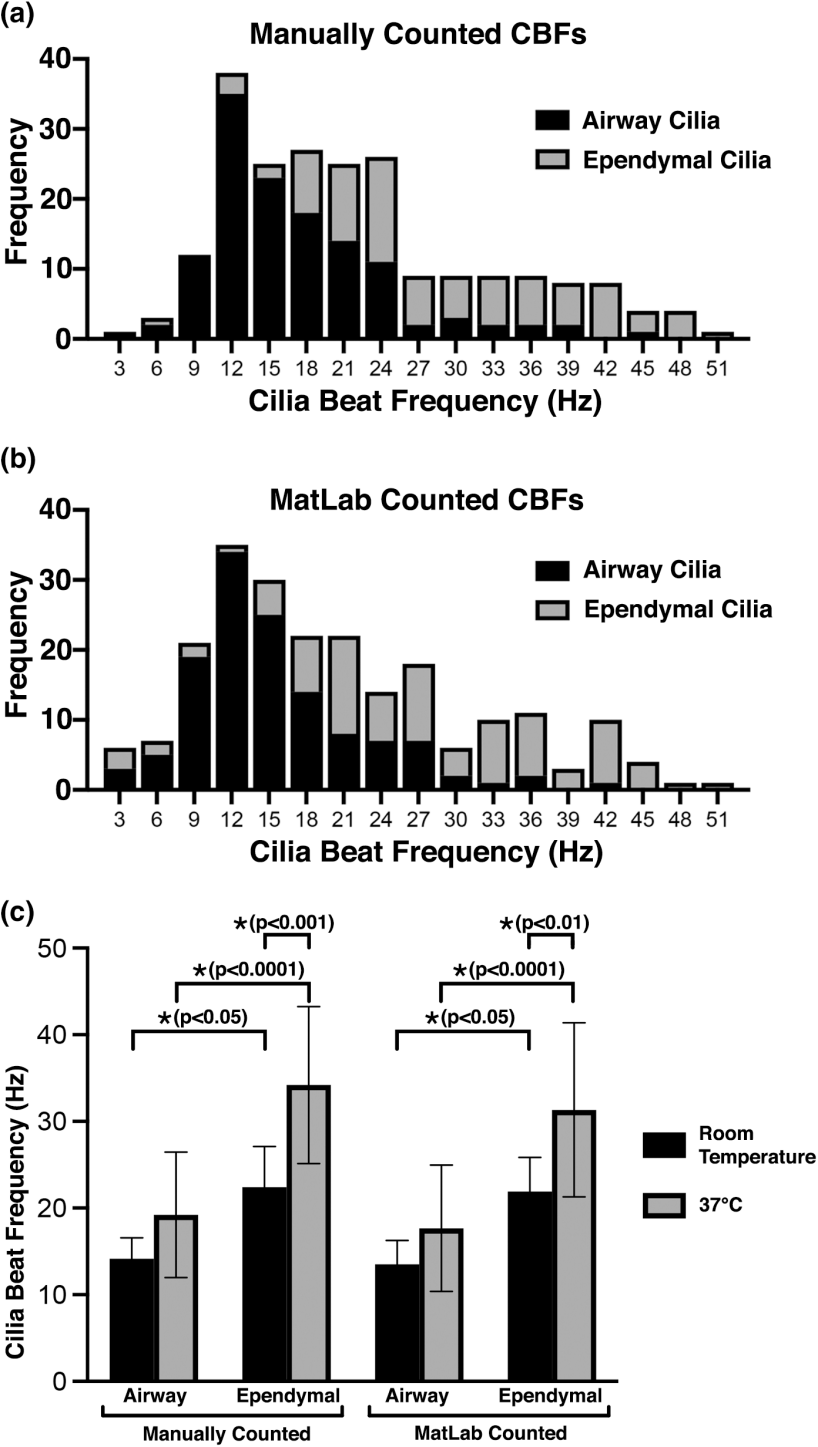
Airway or ependymal cilia beat frequencies as quantified from movies sampled at 300 fps. (a) Frequency distribution of CBFs calculated by manual counting of kymograph peaks generated from movies of airway (*n* = 129) and ependymal cilia (*n* = 105). (b) Frequency distribution of CBFs calculated using an FFT‐based algorithm (MATLAB) to analyze the same kymographs of airway (*n* = 129) and ependymal cilia (*n* = 105) as above. (c) Mean CBFs of airway and ependymal cilia at room temperature and at 37°C, as quantified by manual counting or FFT‐based algorithm. Data presented as mean ± SD. *p*‐values calculated via two‐way ANOVA and post‐hoc Šídák's multiple comparisons test.

Ciliated samples displayed a wide range of calculated CBFs (Figure 1). CBF values manually calculated from airway and ependymal cilia ranged between 4 and 52 Hz, with airway cilia displaying lower CBFs in general than ependymal cilia (Figure 1a). Use of MATLAB to automate CBF calculation from the same raw data resulted in a similar spread of CBFs (between 3 and 52 Hz), with airway cilia also displaying lower CBFs in general than ependymal cilia (Figure 1b). Mean CBF values calculated from the movies sampled at 300 fps gave results as expected from previously published studies (Figure 1c). Elevated incubation temperature (room temperature vs, 37°C) resulted in increased CBFs in all ciliated tissues imaged (14.2 ± 2.4 Hz vs. 19.2 ± 7.2 Hz (*p* = ns) in airways, 22.4 ± 4.7 Hz vs. 34.2 ± 9 Hz (*p* = 0.0002) in ependyma), while ependymal cilia were found to beat significantly faster than airway cilia (22.4 ± 4.7 Hz vs. 14.2 ± 2.4 Hz (*p* = 0.0493) at room temp; 34.2 ± 9.0 Hz vs. 19.2 ± 7.2 (*p* < 0.0001) at 37°C). The method of CBF quantification (Manual counting vs. MATLAB) was found to have no significant influence (*p* > 0.99) on the mean CBF values calculated in either tissue or temperature from the movies sampled at 300 fps (Figure 1c).

### 3.2 Effect of altering movie fps on calculated mean CBFs

To assess how movie fps influenced the calculation of mean CBF, original recordings of cilia motion (>600 fps) were each converted into a set of movies of the same cilia, but with different framerates (300, 200, 150, 100, 60, and 30 fps). Care was taken so that identical regions of identical cilium were analyzed in each movie with differing framerates to calculate CBF so the effect of altering movie fps on CBF could be clearly compared. The gross appearance of cilia motion did not look different within the movies of different framerates when played in real‐time (Movie S2). However, slowing movie playback to 10% real‐time highlights the loss of temporal resolution occurring in lower fps movies (Movie S3).

**FIGURE 2:**
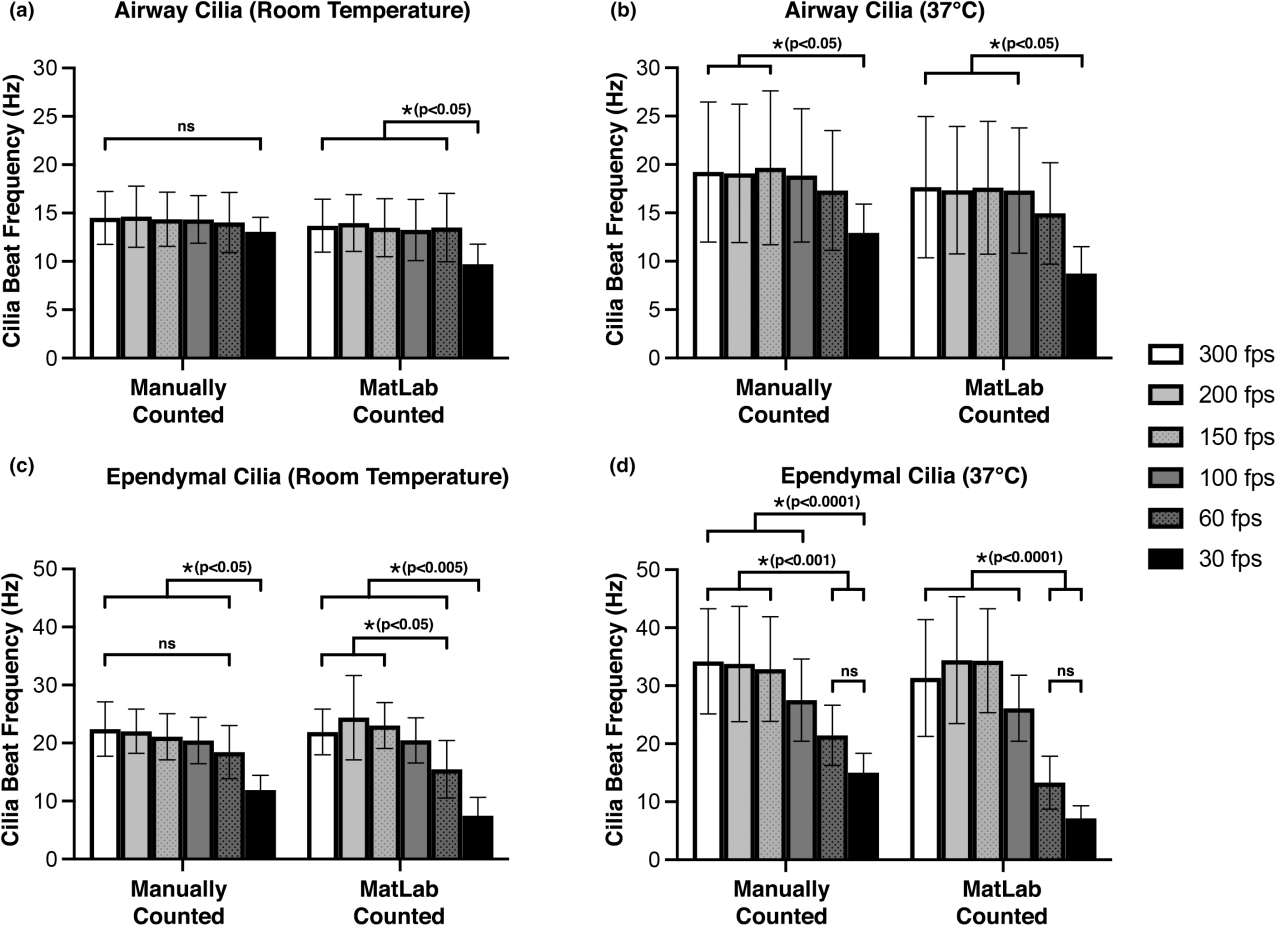
Cilia beat frequency as quantified from movies of differing framerates (30–300 fps) and temperatures (room temperature or 37°C). CBF was quantified by either manual counting or FFT‐based algorithm (MATLAB) as indicated. (a) CBFs of airway cilia imaged at room temperature (*n* = 57). (b) CBFs of airway cilia imaged at 37°C (*n* = 72). (c) CBFs of ependymal cilia imaged at room temperature (*n* = 39). (d) CBFs of ependymal cilia imaged at 37°C (*n* = 66). Data presented as mean ± SD. *p*‐values calculated via two‐way ANOVA and post‐hoc Šídák's multiple comparisons test. Ns = not significantly different (*p* > 0.05).

Our results show that as movie fps was reduced, there was a decrease in calculated mean CBF compared with higher fps movies (Figure 2). This decrease was more marked for cilia incubated at 37°C versus room temp (Figure 2b,d vs. Figure 2a,c), and for ependymal cilia versus airway cilia (Figure 2c,d vs. Figure 2a,b). The largest variation in mean CBF due to altered movie fps was seen in movies of ependymal cilia incubated at 37°C (Figure 2d). Mean ependymal CBFs calculated at 300 fps (34.2 ± 9.1 Hz), 200 fps (33.8 ± 9.9 Hz), and 150 fps (32.9 ± 9.0 Hz) were not significantly different from each other (P > 0.9999); but all were significantly higher (*p* < 0.0001) than ependymal CBFs calculated from movies at 60 fps (21.5 ± 5.2 Hz) and 30 fps (15.0 ± 3.3 Hz). This large variation in calculated CBF observed for ependymal cilia incubated at 37°C is probably due to them displaying the highest average CBF (Figure 1c), with temporal under‐sampling more likely to result in underestimation of their CBF.

Conversely, the smallest variation in mean CBF due to altered movie fps was seen in movies of airway cilia incubated at room temperature (Figure 2a). Mean airway CBFs calculated at 300 fps (14.5 ± 2.7 Hz), 200 fps (14.6 ± 3.2 Hz), 150 fps (14.4 ± 2.8 Hz), 100 fps (14.3 ± 2.5 Hz), 60 fps (14.0 ± 3.1 Hz), and 30 fps (13.1 ± 1.5 Hz) were not significantly different from each other (*p* > 0.9999) when calculated manually. This minimal variation in CBF observed for airway cilia incubated at room temperature is probably due to them having the lowest average CBF (Figure 1c) meaning lower sampling rates would be less likely to result in underestimation of CBFs.

Average CBFs of airway cilia at 37°C and ependymal cilia at room temperature both showed that mean CBF calculated from 30 fps movies was significantly lower (*p* < 0.05) than mean CBFs manually calculated from higher fps movies (Figure 2b,c). There was no significant difference between CBFs calculated for these cilia between 300 and 60 fps (*p* > 0.05), although there was a trend for CBF to be decreased in 60 fps movies versus higher fps movies (Figure 2b,C). This significant reduction in average CBF observed at 30 fps, strongly suggests that sampling rates of >30 fps are required to accurately calculate the CBF of these moderately beating cilia.

The observed decrease in mean CBFs seen in lower fps movies appeared to be magnified if CBF calculation was conducted using an automated FFT‐based approach (MATLAB) as opposed to manual quantification (Figure 2). For example, for ependymal cilia incubated at 37°C (Figure 2d), manually counted CBFs were noticeably higher than FFT‐based calculated CBFs at both 60 fps (21.5 ± 5.2 vs. 13.3 ± 4.6 Hz), and 30 fps (15.0 ± 3.3 vs. 7.2 ± 2.2 Hz). Furthermore, this impairment of the FFT‐based algorithm to accurately calculate CBF was significant in movies of airway cilia incubated at room temperature (Figure 2a). Where the average CBF calculated by FFT‐based algorithm from 30 fps movies (9.7 ± 2.1 Hz) was significantly lower (*p* < 0.05) than the average CBFs calculated by the same algorithm from all higher fps movies (300 fps: 13.7 ± 2.7 Hz; 200 fps: 14.0 ± 2.9 Hz; 150 fps: 13.5 ± 2.9 Hz; 100 fps: 13.3 ± 3.2 Hz; 60 fps: 13.5 ± 3.5 Hz). These results indicate that care should be taken when automating CBF calculation, and if low fps movies are being analyzed to calculate CBF, manual counting may give more accurate results. Conversely, the mean CBFs calculated from all movies >150 fps appeared not noticeably influenced by quantification method (Manual vs. MATLAB) (Figure 2), indicating that automating CBF calculation is accurate if the sample rate is high enough.

### 3.3 Pairwise assessment of the relationship between movie framerate and calculated CBF

**FIGURE 3:**
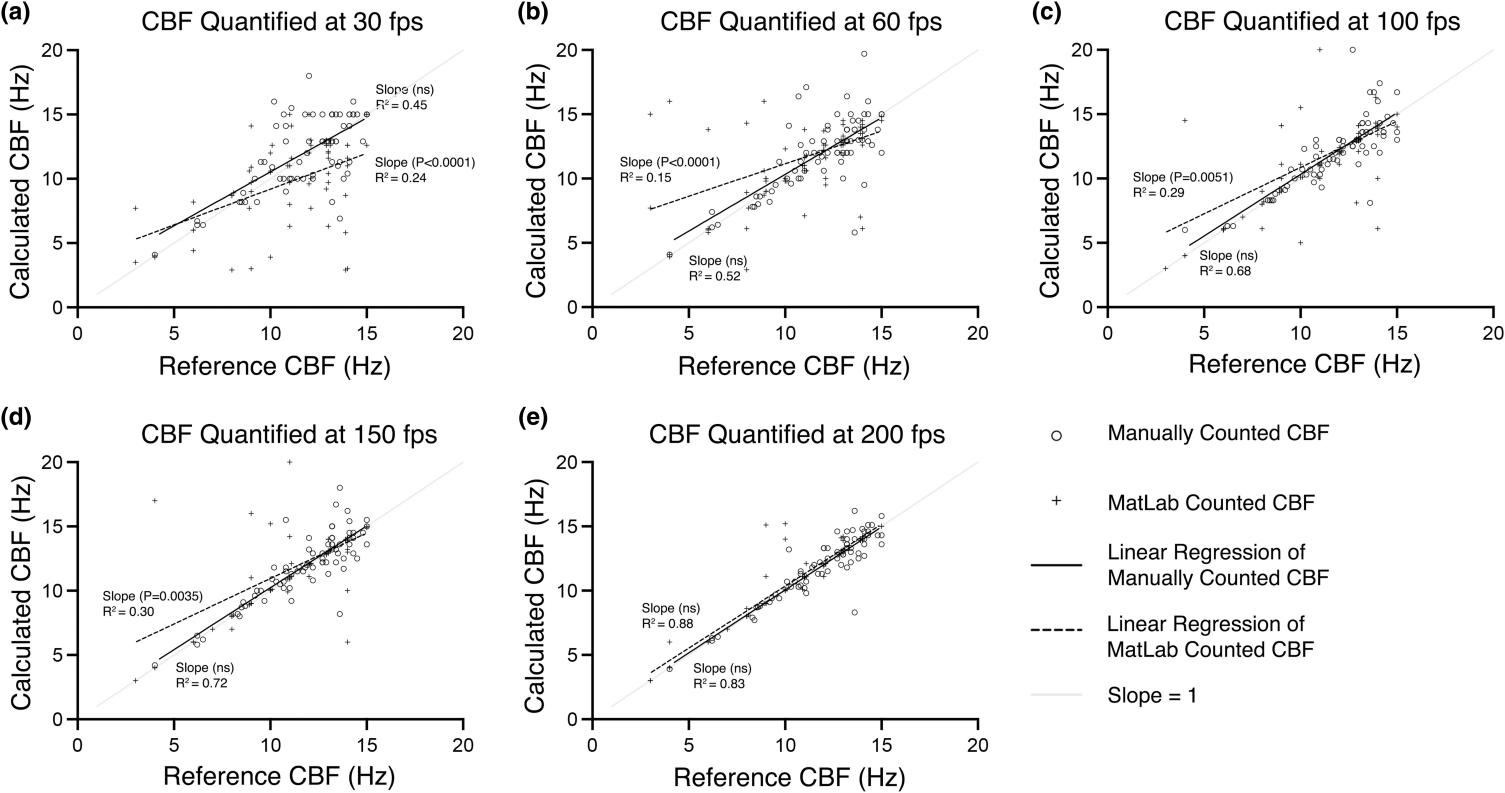
Assessing how movie framerate influences CBF measurement accuracy for cilia beating between 0–15 Hz, as quantified by either manual counting or FFT‐based algorithm (MATLAB). Cilia data from all tissues and temperatures that displayed reference CBFs between 0–15 Hz was pooled (*n* = 73) (reference CBF was calculated from 300 fps movies). Reference CBFs were then compared with the CBFs calculated for the same cilia but from movies with slower framerates (30–200 fps). Linear regression analysis was used to calculate best‐fit lines and R^2^ values for each pairwise comparison dataset. ANCOVA was used to compare best fit lines with an idealized dataset where calculated CBF perfectly matched reference CBF for each cilium (slope = 1). *p*‐values indicate significant divergence of best fit lines from this idealized dataset (i.e., suggesting significant impairment in the accuracy of measuring CBF at that framerate). Ns = not significantly different (*p* > 0.05).

**FIGURE 4:**
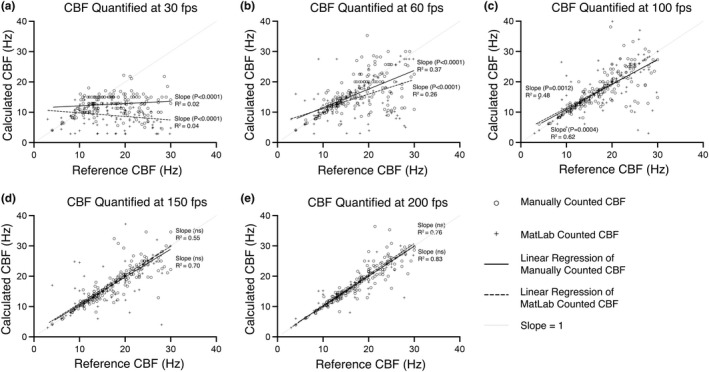
Assessing how movie framerate influences CBF measurement accuracy for cilia beating between 0–30 Hz, as quantified by either manual counting or FFT‐based algorithm (MATLAB). Cilia data from all tissues and temperatures that displayed reference CBFs between 0–30 Hz was pooled (*n* = 171) (reference CBF was calculated from 300 fps movies). Reference CBFs were then compared with the CBFs calculated for the same cilia but from movies with slower framerates (30–200 fps). Linear regression analysis was used to calculate best‐fit lines and R^2^ values for each pairwise comparison dataset. ANCOVA was used to compare best fit lines with an idealized dataset where calculated CBF perfectly matched reference CBF for each cilium (slope = 1). *p*‐values indicate significant divergence of best fit lines from this idealized dataset (i.e., suggesting significant impairment in the accuracy of measuring CBF at that framerate). Ns = not significantly different (*p* > 0.05).

**FIGURE 5:**
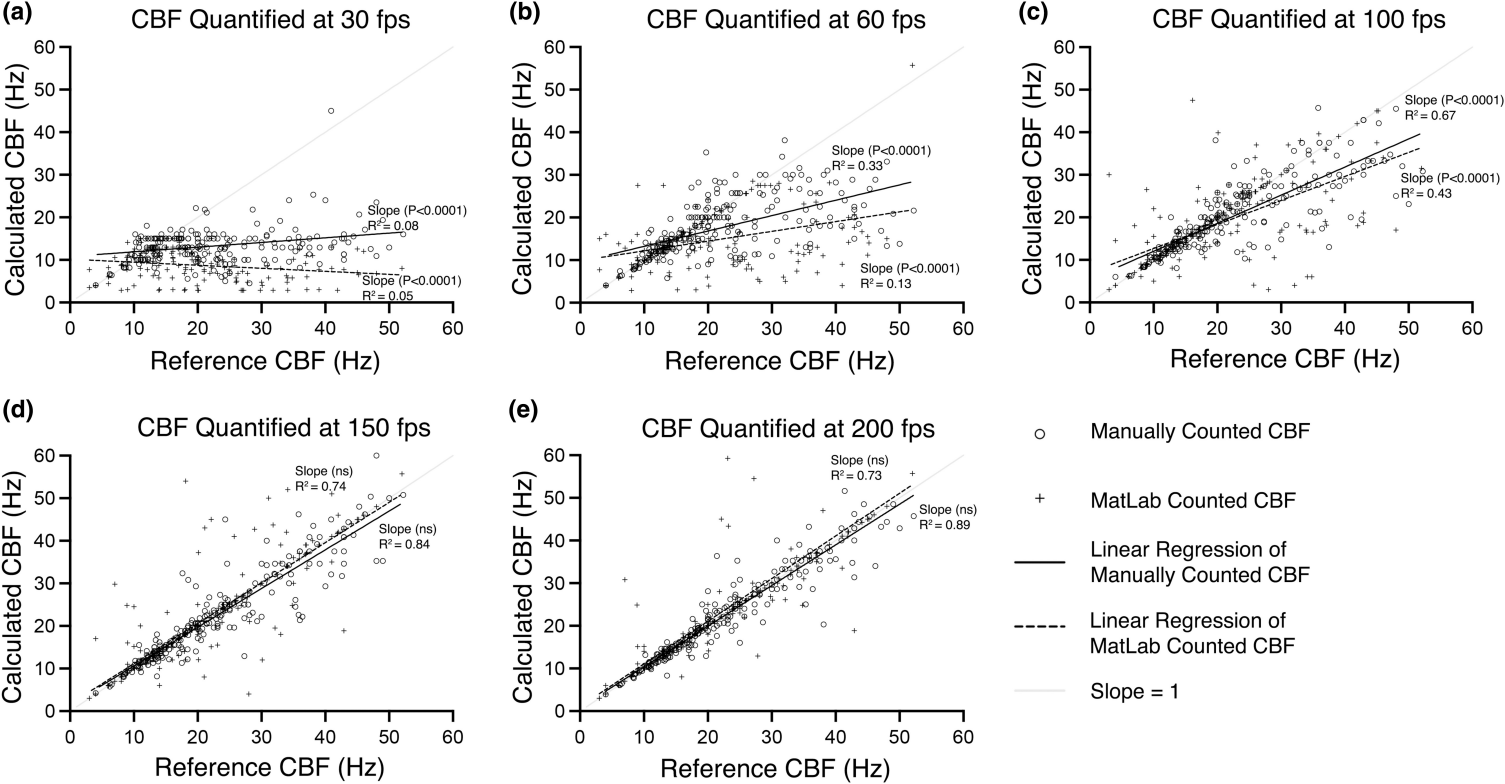
Assessing how movie framerate influences CBF measurement accuracy for cilia beating between 0 and 50 Hz, as quantified by either manual counting or FFT‐based algorithm (MATLAB). Cilia data from all tissues and temperatures that displayed reference CBFs between 0–50 Hz was pooled (*n* = 234) (reference CBF was calculated from 300 fps movies). Reference CBFs were then compared with the CBFs calculated for the same cilia but from movies with slower framerates (30–200 fps). Linear regression analysis was used to calculate best‐fit lines and *R* ^2^ values for each pairwise comparison dataset. ANCOVA was used to compare best fit lines with an idealized dataset where calculated CBF perfectly matched reference CBF for each cilium (slope = 1). *p*‐values indicate significant divergence of best fit lines from this idealized dataset (i.e., suggesting significant impairment in the accuracy of measuring CBF at that framerate). Ns = not significantly different (*p* > 0.05).

CBFs from all tissues and temperatures were pooled into a single dataset (*n* = 234 cilia). CBF values calculated from the 300 fps movies were defined as reference CBFs, then reference CBFs for each cilium were compared in a pairwise fashion with its CBF calculated from lower framerate movies. Pairwise comparisons were conducted using three subsets of the pooled CBF data, reference CBFs beating between 0 and 15 Hz (Figure 3), reference CBFs beating between 0 and 30 Hz (Figure 4), and reference CBFs beating between 0 and 50 Hz (Figure 5). These three subsets of CBFs were chosen to aid in delineating the relationship between movie fps and CBF measurement accuracy, and to test if Nyquist sampling (i.e., sampling frequency = double data frequency) could be used to determine the optimum movie fps for calculating CBF. If Nyquist sampling could be used to determine optimal sampling fps, CBFs for cilia beating between 0 and 15 Hz could be accurately determined from 30 fps movies (Figure 3), and CBF for cilia beating between 0 and 30 Hz could be accurately determined from 60 fps movies (Figure 4).

For cilia beating between 0–15 Hz (Figure 3; *n* = 73), the best fit line for manually counted CBFs appeared not significantly influenced by any movie fps analyzed (i.e., best fit line slopes were not significantly different from reference slope at any fps) (Figure 3a–e). However, *R* ^2^ values for manually counted CBFs were found to be noticeably influenced by movie fps, with variability decreasing as fps increased (30 fps: *R* ^2^ = 0.45; 60 fps: *R* ^2^ = 0.52; 100 fps: *R* ^2^ = 0.68; 150 fps: *R* ^2^ = 0.72; 200 fps: *R* ^2^ = 0.83). Conversely, for cilia beating between 0 and 15 Hz, the best fit line for FFT‐based algorithm quantified CBFs was found to be significantly influenced by movie fps, with best fit line slopes being significantly different from the reference slope at all framerates except 200 fps (Figure 3a–e). *R* ^2^ values for FFT‐based algorithm quantified CBFs showed a similar trend to those for the manually counted data, with data variability decreasing as fps increased (30 fps: *R* ^2^ = 0.24; 60 fps: *R* ^2^ = 0.15; 100 fps: *R* ^2^ = 0.29; 150 fps: *R* ^2^ = 0.30; 200 fps: *R* ^2^ = 0.88). Data variability (*R* ^2^) was markedly greater for FFT‐based algorithm quantified CBFs than manually counted CBFs at all movie fps except 200 fps (Figure 3).

For cilia beating between 0 and30 Hz (Figure 4; *n* = 171), the best fit line for manually counted CBFs appeared significantly influenced by movie fps at 30 fps (*p* < 0.0001), 60 fps (*p* < 0.0001), and 100 fps (*p* = 0.0004) (Figure 4a–c); while best fit lines were not significantly influenced by movies at 150 and 200 fps (Figure 4d,e). The *R* ^2^ values for manually counted CBFs within this group demonstrated that data variability decreased as fps increased (30 fps: *R* ^2^ = 0.02; 60 fps: *R* ^2^ = 0.37; 100 fps: *R* ^2^ = 0.62; 150 fps: *R* ^2^ = 0.70; 200 fps: *R* ^2^ = 0.83). FFT‐based algorithm quantified CBFs for cilia beating between 0 and 30 Hz showed a similar trend to that of the manually counted CBF data. Namely, best fit lines for FFT‐based algorithm quantified CBFs appeared significantly influenced by movie fps at 30 fps (*p* < 0.0001), 60 fps (*p* < 0.0001), and 100 fps (*p* = 0.0012) (Figure 4a–c); while best fit line slopes were not significantly influenced by movies at 150 and 200 fps (Figure 4d,e). The *R* ^2^ values for FFT‐based algorithm quantified CBFs demonstrated a similar reduction in data variability as fps increased (30 fps: *R* ^2^ = 0.04; 60 fps: *R* ^2^ = 0.26; 100 fps: *R* ^2^ = 0.48; 150 fps: *R* ^2^ = 0.55; 200 fps: *R* ^2^ = 0.76).

Finally, best fit lines for manually counted CBFs for cilia beating between 0 and 50 Hz (Figure 5; *n* = 234) appeared similar to those seen for cilia beating between 0 and 30 Hz (Figure 4). Namely, best fit lines appeared significantly influenced by movie fps at 30 fps (*p* < 0.0001), 60 fps (*p* < 0.0001), and 100 fps (*p* < 0.0001) (Figure 5a–c), while best fit lines were not significantly influenced by movies at 150 and 200 fps (Figure 5d,e). *R* ^2^ values for this data also demonstrated data variability decreased as movie fps increased for manually counted CBF (30 fps: *R* ^2^ = 0.08; 60 fps: *R* ^2^ = 0.33; 100 fps: *R* ^2^ = 0.67; 150 fps: *R* ^2^ = 0.84; 200 fps: *R* ^2^ = 0.89). FFT‐based algorithm quantified CBFs for cilia beating between 0 and 50 Hz showed a similar trend to that of the manually counted CBF data (Figure 5). Namely, best fit lines for FFT‐based algorithm quantified CBFs appeared significantly influenced by movie fps at 30 fps (*p* < 0.0001), 60 fps (*p* < 0.0001), and 100 fps (*p* < 0.0001) (Figure 5a–c); while best fit line slopes were not significantly influenced by movies at 150 and 200 fps (Figure 5d,e). *R* ^2^ values for FFT‐based algorithm quantified CBFs demonstrated a similar reduction in data variability as fps increased (30 fps: *R* ^2^ = 0.05; 60 fps: *R* ^2^ = 0.13; 100 fps: *R* ^2^ = 0.43; 150 fps: *R* ^2^ = 0.74; 200 fps: *R* ^2^ = 0.73).

## 4 DISCUSSION

Motile cilia are found in numerous locations throughout our bodies and their motility underlies various important physiological processes (Kumar et al., 2021; Legendre et al., 2021; Wallmeier et al., 2020). The easiest way to assess motile cilia function is by quantifying CBF via highspeed video microscopy (Francis & Lo, 2013; O'Callaghan et al., 2012; Sisson et al., 2003) and this forms a sizable chunk of the cilia literature. However, no standardized frame rate exists when imaging cilia motility for subsequent CBF calculation, this has resulted in a large range of frames rates used to image cilia motility within the published literature (Abdelhamed et al., 2018; Abdelhamed et al., 2020; Bustamante‐Marin et al., 2019; Chen et al., 2016; Hagiwara et al., 2020; Hennessy et al., 1986; Liu et al., 2021; Mateos‐Quiros et al., 2021; Mikhailik et al., 2021; Reula et al., 2021; Smith et al., 2012; Zahid et al., 2020). The lack of a standardized fps for imaging motile cilia is especially problematic because it hinders high‐speed video microscopy from being clinically adapted as a diagnostic tool to help uncover motile cilia diseases, such as primary ciliary dyskinesia (Bricmont et al., 2021; Shapiro et al., 2018). The large disparity in frame rates used to image cilia motility is partly technology dependant, with early studies generally being restricted to lower fps imaging technology (2–25 Hz) (Clary‐Meinesz et al., 1992; Hennessy et al., 1986), while more recent studies with access to much faster and cheaper imaging technology generally having higher fps (≥500 fps) (Smith et al., 2012; Yasuda et al., 2020). Thus, the need exists to determine the optimum frame rate needed for imaging cilia using video microscopy for subsequent CBF calculation.

The present study provides a large dataset of motile cilia recordings from different cilia populations displaying a wide range of CBF values. Our data matches previously published findings by highlighting the significant difference in CBF between airway and ependymal cilia and by emphasizing how CBF is modulated by changes in temperature. Murine ependymal cilia have been previously shown to beat significantly faster than airway cilia (Francis et al., 2009; Hagiwara et al., 2020; Mahuzier et al., 2018; Mikhailik et al., 2021), while increased incubation temperature is well recognized to result in a linear increase in mean CBF (Christopher et al., 2014; Schipor et al., 2006; Sisson et al., 2003; Welchering et al., 2015).

In the first part of this study, we compared mean CBF values generated for identical cilia within movies of different sampling rates (fps) (Figure 1). Our data demonstrates that for populations of slower cilia, slower movie sampling rates appear adequate for quantifying CBF manually. For example, room temperature airway cilia were the slowest imaged, and their CBF could be manually calculated from 30 fps movies without apparent significant impairment. Conversely, for faster cilia populations, we found that faster movie sampling rates are essential to successfully quantify CBF. For example, airway cilia at 37°C displayed a significant impairment in mean CBF when manually calculated from movies sampled at 30 fps. Measurement of mean CBF from slower sampled movies was also impaired in ependymal cilia at both room temperature and 37°C, while CBF of ependymal cilia at 37°C displayed significant impairment when manually calculated from movies at both 60 and 30 fps.

**FIGURE 6:**
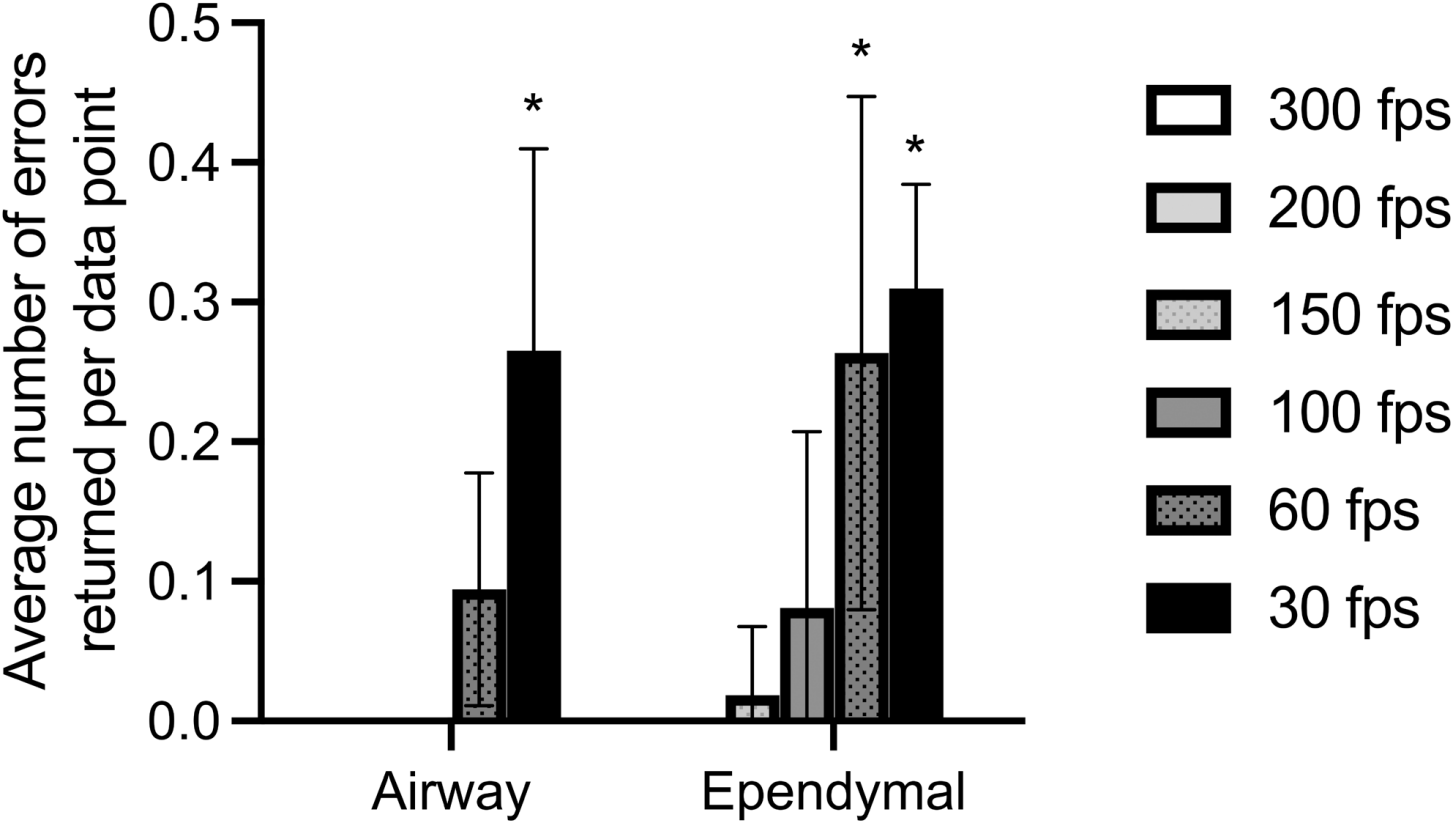
Failure rate of MATLAB to calculate CBF in airway versus ependymal epithelia as influenced by movie framerate. Data presented as mean ± SD. *p*‐values calculated via two‐way ANOVA and post‐hoc Šídák's multiple comparisons test. * = *p* < 0.05.

We also found in the first part of this study, that CBF quantification method (Manual vs. FFT‐based algorithm in MATLAB) also significantly influenced the effect sampling rate had on calculated mean CBF accuracy. This was perhaps best highlighted by the room temperature airway cilia, where cilia movies sampled at 30 fps appeared adequate to manually count CBF without apparent significant impairment; while the same 30 fps movies when used to quantify CBF using an automated FFT‐based algorithm (MATLAB) displayed a significant impairment in mean CBF (Figure 1a). A general trend for calculating lower CBF values were seen when an FFT‐based algorithm was used to analyze slower sampled movies. However, this inaccuracy disappeared when the FFT‐based algorithm was used to calculate CBF from faster sampled movies (100–150 fps). The influence of movie fps on the ability of the FFT‐based algorithm to successfully quantify CBF is highlighted in Figure 6. Figure 6 shows that the average number of calculation errors returned by FFT‐based algorithm per cilia kymograph significantly increases as movie fps decreases. We found that the FFT‐based algorithm had problems calculating CBF for close to 30% of airway cilia imaged at 30 fps and close to 30% of ependymal cilia imaged at both 30 and 60 fps. The apparent higher accuracy of CBFs calculated manually versus calculated by FFT‐based algorithm from lower fps movies is not surprising as the human brain is well known for its superlative pattern recognition (Mattson, 2014) which would make it excellent for quantifying CBF from noisy cilia kymographs (Figure S1). While the MATLAB script utilized a common technique for analyzing CBF (i.e., fast Fourier transform) (Awatade et al., 2021; Lindberg et al., 1996; Reula et al., 2021; Sisson et al., 2003; Smith et al., 2012), it may be possible to refine the automated analysis to provide more accuracy at lower fps movies. A possible solution for automated analysis for data collected with low framerates, is to employ the spatio‐temporal pixels' cross‐correlation over varying field of view centred at ciliary region (Chioccioli et al., 2019) to compensate for the loss in temporal resolution. However, this would need further testing in future studies and may be unnecessary if all that is required to ensure accuracy using an FFT‐based algorithm is to avoid imaging cilia at too slow a framerate.

In the second part of the study, we pooled all CBF data into three datasets as defined by the CBF values calculated from the 300 fps movies, which we defined as the reference CBFs (the assumption being that the CBFs calculated from the fastest fps movies as being the most accurate). The three pooled datasets included cilia with reference CBFs between 0 and 15 Hz (Figure 3), cilia with reference CBFs between 0–30 Hz (Figure 4), and cilia with reference CBFs beating between 0 and 50 Hz (Figure 5). The reference CBF for each cilium within each dataset was then compared in a pairwise fashion with the CBF calculated for the same cilium from the lower framerate movies to assess the relationship between CBF measurement accuracy and movie framerate. The rationale for pooling cilia data into these three datasets was to assess if the Nyquist theorem could be used as a general rule for determining adequate sampling rate for accurate CBF quantification from cilia movie data. The Nyquist theorem specifies that to analyze sinusoidal data with no loss of information, sampling frequency should be twice the sinusoidal frequency (Colarusso et al., 1999; Oshana, 2006). Thus, if the Nyquist theorem was applicable for determining optimal movie fps for measuring CBF from cilia movies, cilia within the 0–15 Hz dataset would be accurately quantified by ≥30 fps movies, cilia within the 0–30 Hz dataset would be accurately quantified by ≥60 fps movies, while cilia within the 0–50 Hz dataset would only be accurately quantified by ≥100 fps movies.

Our data shows that for slower cilia (0–15 Hz), manual counting of CBF from 30 fps movies appeared to provide an accurate assessment of CBF as shown by pairwise liner regression, which was not significantly different from the reference line (Figure 3). However, FFT‐based algorithm calculation of CBF for the same slower cilia appeared significantly impaired versus manual counting at all fps except at 200 fps. This data suggests that while the Nyquist theorem may be useful to determine optimum sampling fps for manual quantification of CBF for slow cilia (0–15 Hz), the Nyquist theorem may not be useful when using automated quantification of CBF via FFT‐based algorithm. Paradoxically, for the other two faster cilia datasets (0–30 Hz & 0–50 Hz), both manual and the FFT‐based algorithm calculated CBFs were only accurate in movies sampled at ≥150 fps (Figures 4, 5). This suggests that the Nyquist theorem is not useful for determining optimum sampling fps for populations of cilia including medium (0–30 Hz) to fast cilia (0–50 Hz) when quantifying CBF using either manual counting or FFT‐based algorithm. Thus, while the Nyquist theorem may appear a logical way for setting movie fps when imaging cilia for subsequent CBF calculation, our data suggests that video microscopy of ciliated in vitro tissues is associated with too much noise to make this practical. To counteract this, we suggest that the sampling rate (fps) should be at least four times that of the expected fastest CBF within the tissue being measured (i.e., double the value predicted by the Nyquist theorem).

A similar study was conducted by Chen et al which assessed the effect of different sampling rates (30, 60, 120, 240 fps) on calculated CBF from rabbit trachea samples at room temperature and 37°C (Chen et al., 2016). Chen et al found that both manual counting and FFT‐based algorithm quantification provided accurate assessment of CBF at the lowest fps (30 fps) (Chen et al., 2016). However, their calculated CBFs were quite low (~9 Hz @ 37°C) which matches that found in this study for manually counted CBF from slow cilia populations. Their observation that FFT‐based algorithm calculated CBF at 30 fps was accurate may reflect the slower CBF they recorded (~9 vs. ~14 Hz for the slowest cilia in this study), or possibly a difference in the FFT‐based algorithm they used.

A wide variety of different published methods exist for quantification of CBF from microscopy recordings, including the use of MATLAB (Awatade et al., 2021; Chen et al., 2016; Mateos‐Quiros et al., 2021), LabVIEW (Sisson et al., 2003), and ImageJ plugins (Smith et al., 2012). While we did not access the effect of movie fps on CBF quantification using these other software methods, we suggest that if they are to be used, researchers should properly determine how they are influenced by movie fps. Our observations show that FFT‐based algorithm does provide accurate measurements of CBF if movie fps is set high enough (≥150 fps), but care should be taken when calculating CBF from lower fps movies. If low frame rate movies are all that is available, then manual counting may provide a more accurate assessment of CBF depending on how fast the cilia being recorded are beating.

Finally, it should be stressed that this study was designed to determine the influence of movie fps on CBF measurement. For cilia beat pattern analysis (e.g., normal cilia motion vs. dyskinetic cilia motion) higher fps movies (i.e., ≥500 Hz) may be required to fully observe the fine movements of cilia (Yasuda et al., 2020).

## 5 CONCLUSION

In conclusion, we found that when CBF is being manually quantified for slower cilia populations (≤15 Hz) the Nyquist theorem may be used to set video microscopy sampling rate (fps). However, for faster cilia populations (0–30 Hz or 0–50 Hz), or when CBF is being calculated using an FFT‐based algorithm, the Nyquist theorem appears unreliable for setting video microscopy sampling rate, and we suggest that sampling rate (fps) should be at least 3–4 times that of the expected fastest CBF within the tissue being measured for accurate CBF assessment.

## Supporting information




Data S1
