# Supplementary material for: Quantifying cilia beat frequency using high‐speed video microscopy: Assessing frame rate requirements when imaging different ciliated tissues
Source: Physiol Rep. 2022 Jun 8;10(11):e15349. doi: 10.14814/phy2.15349 (PMC9178357; doi:10.14814/phy2.15349)
Supplement: Supplementary file 1 — Data S1 [file PHY2-10-e15349-s001.docx]

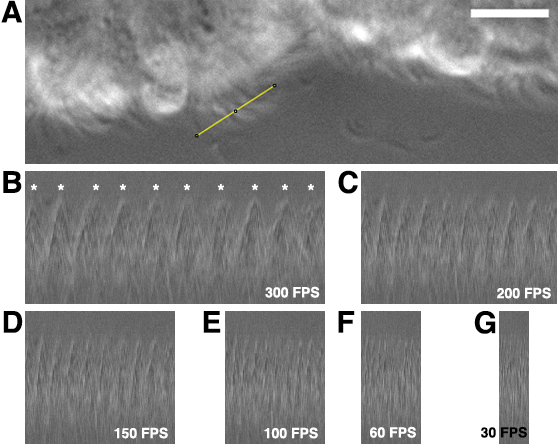


**Supplementary Figure 1.** Example outlining how kymographs were generated from cilia recordings to quantify CBF at varying framerates. A one second movie of cilia motility was first collected at >600 fps, this initial recording was used to generate movies of the same field of view at 300, 200, 150, 100, 60, and 30 fps. Kymographs of cilia motility were generated from each movie in ImageJ by using the line tool to draw a line through the cilia of a single ciliated cell (A). This line was then ‘Resliced’ in ImageJ to generate a kymograph image. Each wave peak (*) on a kymograph corresponds to cilia passing through the drawn line. Identical lines were applied to each movie with a different framerate using the ImageJ command ‘Restore Selection’ to generate kymographs of the same cilia at varying sampling framerates (30-300 fps) (B-G). As each movie was one second long, the total number of peaks per kymograph = cilia beat frequency (Hz). Scale Bar = 10 µm.


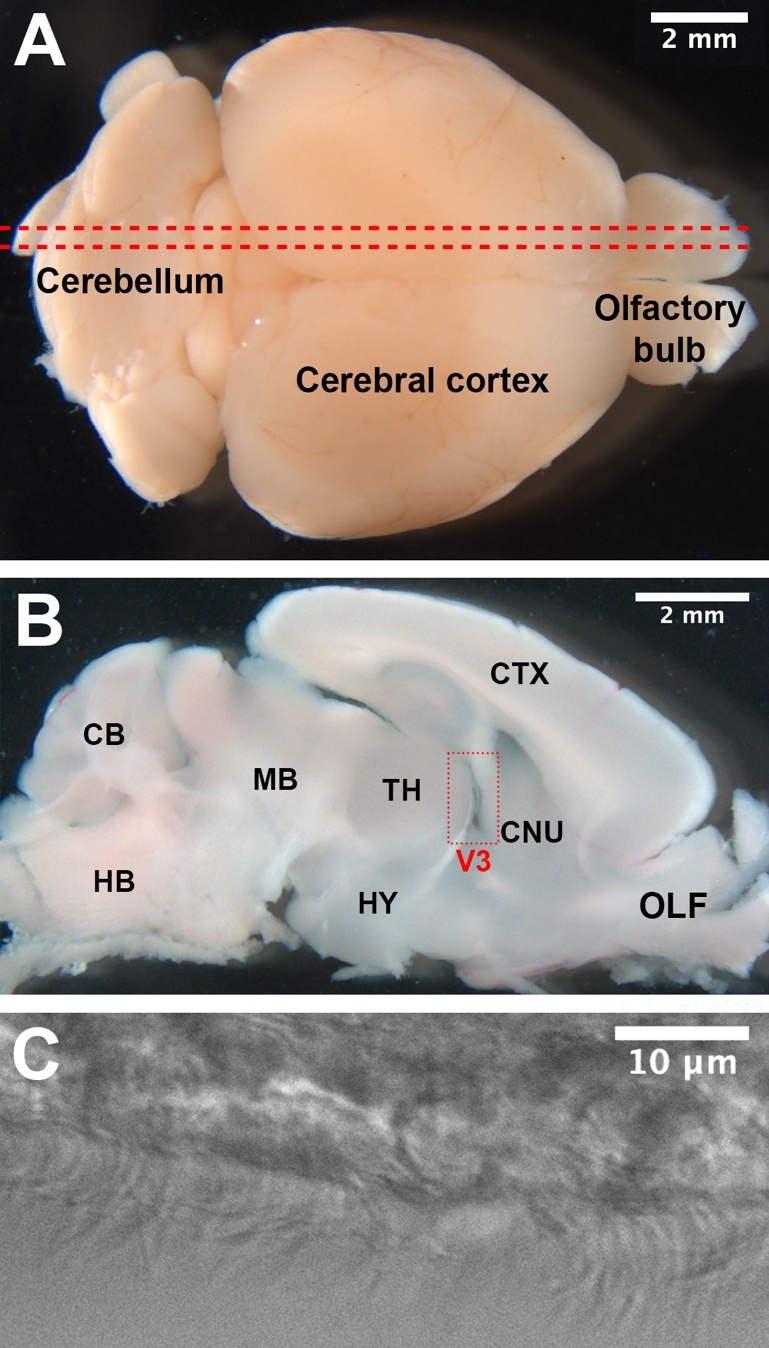


**Supplementary Figure 2.** Example of mouse brain sample preparation for imaging ependymal cilia. A 0.5mm thick sagittal section is obtained using a sagittal brain matrice (red dotted lines in A). Ependymal cilia within the third ventricle are then imaged in each sagittal section (red dotted square in B). Representative image of ependymal cilia visualised in these samples (C). CB: Cerebellum; CNU: Cerebral nuclei; CTX: Cerebral cortex; HB: Hindbrain; HY: Hypothalamus; MB: Midbrain; OLF: Olfactory areas; TH: Thalamus; V3: Third ventricle.


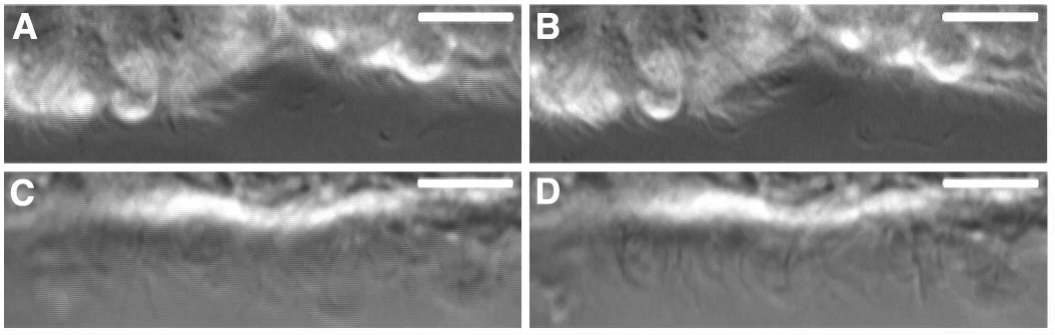


**Supplementary Movie 1.** Representative examples of mouse airway and ependymal cilia motility. (A,B) Airway cilia motility in real-time (A) and slowed to 10% of real-time (B). (C,D) Ependymal cilia motility in real-time (A) and slowed to 10% of real-time (B). Scale Bars = 10 µm.


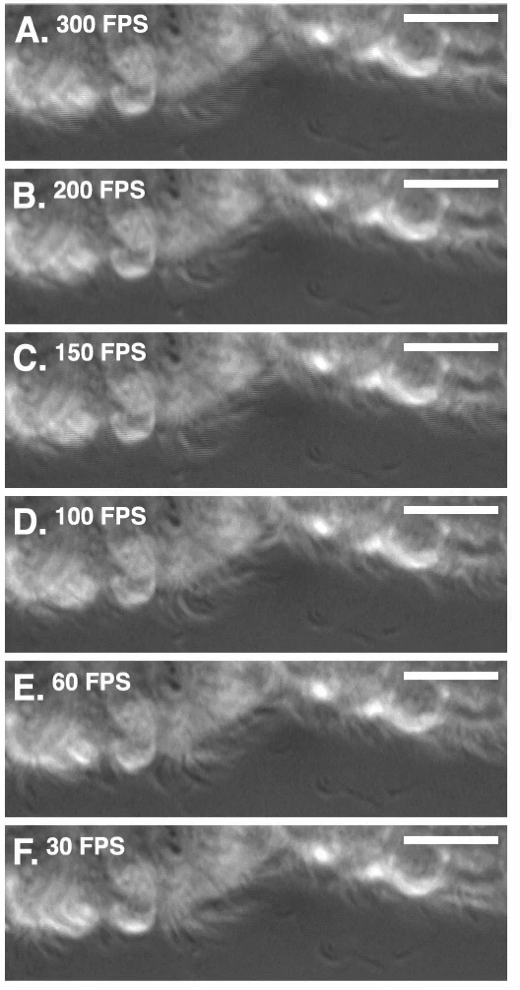


**Supplementary Movie 2.** Demonstration that the gross appearance of cilia motion does not appear effected by movie frame rate (30-300 fps) when observed in real-time. Cilia in this field of view are beating ~10 Hz. Scale Bars = 10 µm.


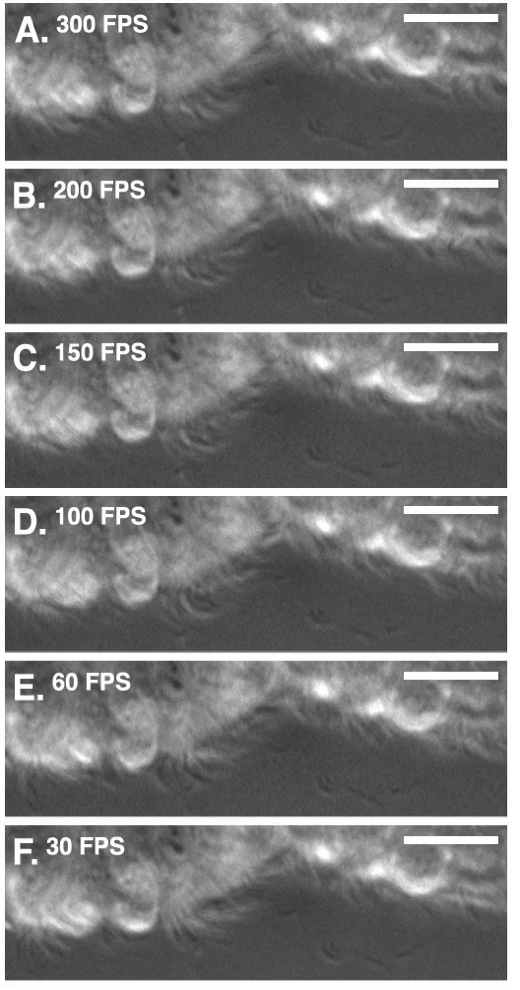


**Supplementary Movie 3.** Demonstration that a significant loss in temporal resolution becomes evident when movies collected at different framerates are slowed to 10% of real-time. Cilia in this field of view are beating ~10 Hz. Scale Bars = 10 µm.
